# RNA Modifications in Tumor Microenvironment: A New Dimension for Cancer Treatment

**DOI:** 10.1002/mco2.70816

**Published:** 2026-06-23

**Authors:** Qiwen Li, Yating Sun, Shuibin Lin, Quan Yuan

**Affiliations:** ^1^ State Key Laboratory of Oral Diseases, National Center for Stomatology, and National Clinical Research Center for Oral Diseases West China Hospital of Stomatology Sichuan University Chengdu Sichuan China; ^2^ Department of Genetics School of Life Sciences Anhui Medical University Hefei Anhui China; ^3^ Center for Translational Medicine Precision Medicine Institute The First Affiliated Hospital Sun Yat‐sen University Guangzhou China; ^4^ State Key Laboratory of Oncology in South China Sun Yat‐sen University Cancer Center Guangzhou China

**Keywords:** immune cells, RNA metabolism, RNA modifications, tumor microenvironment

## Abstract

RNA modifications are pivotal posttranscriptional gene expression regulators with profound functions in both physiological and pathological states, particularly during cancer initiation and progression. Within the complex tumor microenvironment (TME), these modifications orchestrate diverse mechanisms that significantly impact tumor growth, facilitate immune evasion, and drive therapeutic resistance. Recent advances have demonstrated their critical roles in shaping RNA metabolism and maintaining genome integrity, as well as modulating key stromal pathways such as immune responses, extracellular matrix remodeling, and vascular supply. Emerging evidence further suggests that RNA modifications dynamically regulate intercellular communication between tumor cells and surrounding stromal and immune cells within the TME. In this review, we provide a comprehensive overview of the mechanisms and functional roles of major RNA modifications, including adenosine, guanosine, and pseudouridine modifications. We discuss their regulatory roles in RNA metabolism and genome integrity, and examine their diverse functions within the TME, including immune regulation, tumor proliferation, extracellular matrix remodeling, and DNA damage response (DDR) pathways. Additionally, we highlight recent advances in therapeutic strategies targeting RNA‐modifying enzyme and discuss their potential for improving cancer treatment. Collectively, targeting RNA modification represents a promising strategy for overcoming cancer resistance and improving clinical treatment outcomes.

## Graphical abstract

Tumor initiation and progression depend on a supportive and highly heterogeneous tumor microenvironment (TME). RNA modifications regulate signaling pathways and cytokine networks that shape TME dynamics, facilitating interactions between cancer cells and noncancerous stromal cells, and ultimately promoting immune evasion and tumor progression.

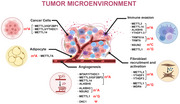

## 1 Introduction

The concept of the “epitranscriptome” has revolutionized our understanding of posttranscriptional gene regulation. RNA modifications have been extensively investigated since their initial discovery, with more than 170 types of chemical modifications identified on various RNA molecules, which exerts significant roles in diverse physiological pathways [1]. Among the RNA modifications identified to date, *N* ^6^‐methyladenosine (m^6^A) is one of the most abundant and most extensively studied modifications in eukaryotes [2]. Additionally, other modifications such as *N* ^1^‐methyladenosine (m^1^A), *N* ^7^‐methylguanosine (m^7^G), *N* ^5^‐methylcytosine (m^5^C), *N* ^3^‐methylcytidine (m^3^C), and pseudouridine (Ψ) are found in both mRNA and noncoding RNAs [3, 4, 5]. Modified RNA molecules play pivotal roles in the regulation of diverse physiological pathways, particularly within complex regulatory networks, where the interplay between signaling and modifications modulate cellular functions [6, 7].

The RNA modification processes are dynamically mediated by various regulators, including methyltransferases (writers), demethylases (erasers), and reading proteins (readers). Writers catalyze the addition of RNA modifications, while erasers remove these marks, ensuring the reversibility of these regulatory events. Readers recognize modified RNA bases and relay this information to regulate critical RNA processes, such as maturation and degradation, by activating essential downstream signaling. The dynamic regulation of different enzymes is essential for maintaining an overall balance of RNA modifications. Dysregulation of RNA modifications often contribute to tumor initiation and metastasis, while tumor cells exploit these mechanisms to achieve immune evasion and drug resistance [8, 9, 10].

While cancer cells have been extensively studied, the broader impact of RNA modifications on the tumor microenvironment (TME) requires further clarification. The TME represents a highly organized niche comprising cancer cells surrounded by diverse nonmalignant cell types. The noncancerous cellular components include immune cells, cancer‐associated fibroblasts (CAFs), endothelial cells (ECs), pericytes, and tissue‐specific cell types such as adipocytes and neurons [11]. The TME, initially considered bystanders, now appears to play a crucial role in tumorigenesis. The cellular composition and functional status of TME can change dynamically depending on the cancer type and stage, with different cell populations exhibiting either anti‐tumorigenic or tumor promoting functions [12]. The intricate interaction and differential regulation of target genes by RNA modifications in the TME also play a vital role in cell proliferation, growth promotion, and metastasis. Numerous studies have demonstrated the regulatory role of RNA modifications in the TME, contributing to various stages of tumor progression [6].

In this review, we provide a comprehensive overview of the emerging roles of RNA modifications within the TME, following a logical sequence from molecular mechanisms to clinical applications. First, we summarize the regulatory machinery of key modifications, focusing on adenosine, guanosine, and pseudouridine. Second, we discuss their fundamental roles in regulating RNA metabolism and maintaining genome integrity. Third, we systematically examine their diverse functions in modulating specific TME components, including their impact on immune regulation, extracellular matrix (ECM) remodeling, and DNA damage response (DDR) pathways. Finally, we highlight the translational potential of targeting these epitranscriptomic regulators as a novel therapeutic strategy to overcome resistance and improve cancer treatment outcomes.

## 2 Regulation of RNA Modification

**FIGURE 1:**
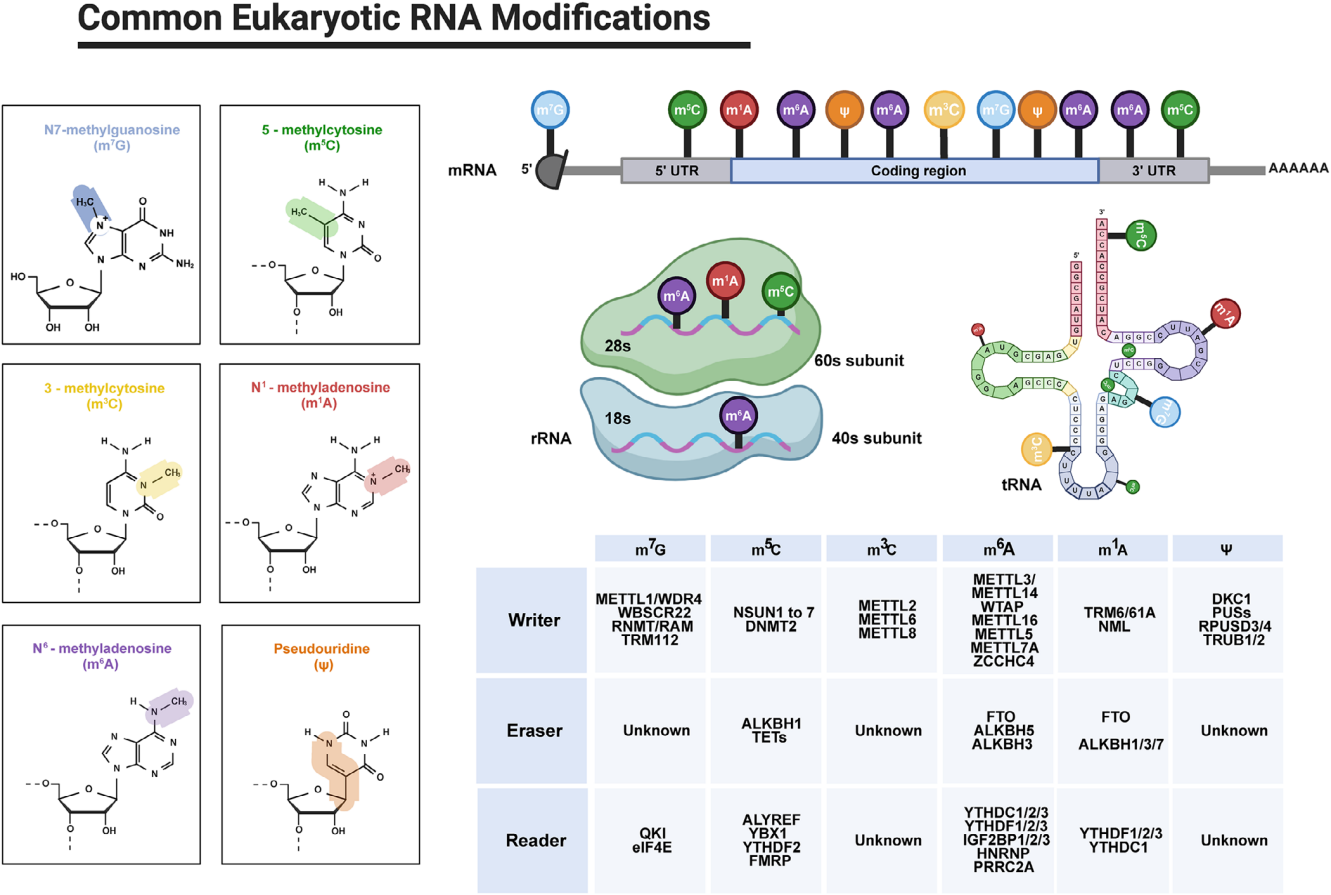
RNA modifications refer to structural alterations on an RNA molecule that modify the canonical AUGC bases. These modifications can be classified into adenosine methylations, cytosine methylations, guanosine methylations, and isomerization of uridine based on their specific locations within the bases. The objective of this image is to provide a comprehensive overview of regulation and regulators involved in RNA modifications across these four classifications. This image was created with BioRender (https://biorender.com/).

RNA modifications refer to structural alterations made to an RNA molecule that modify the canonical AUGC bases. These modifications are not limited to mRNA, tRNA, and ribosomal RNA (rRNA), but are also found in various classes of noncoding RNAs. The reversible RNA modifications, such as methylation and pseudouridylation, actively regulate diverse biological processes, playing crucial role in gene expression and cell function [9, 13]. Studies have reported the active role of several RNA modifications in TME. Collectively, these chemical marks constitute a complex epitranscriptomic network that governs RNA fate. In this section, we systematically introduce the distinct types of RNA modifications, including adenosine, cytosine, guanosine, and uridine modifications in TME regulation. Meanwhile, the enzymes that regulate the dynamic modifications are discussed (Figure 1).

### 2.1 Adenosine Methylation (m^6^A, m^1^A)

The methylation at *N* ^6^‐position of adenosine, known as *N^6^*‐methyladenosine (m^6^A), is the most abundant internal modification on eukaryotic mRNA, accounting for approximately 50% of total methylated ribonucleotides [1]. It has been identified in mRNA [14, 15], rRNA [16], primary miRNA (pri‐miRNA) [17], and long intergenic ncRNAs (lincRNAs) [18]. Extensive research has revealed numerous proteins involved in regulating m^6^A modifications. These include m^6^A methyltransferases, such as methyltransferase‐like (METTL)3, METTL14, METTL16, and WTAP; demethylases including FTO and ALKBH5; and m^6^A‐binding proteins, such as YTHDF1/2/3, YTHDC1/2, IGF2BP1/2/3, and HNRNPA2B1 [19, 20]. For a more detailed discussion on m^6^A modification, please refer to other excellent reviews in this field [21, 22, 23, 24]. On the other hand, m^1^A modification is located at *N*_1_ of adenosine in RNA. It has been found in tRNAs [25], rRNAs [26], mRNAs [27], and long noncoding RNAs (lncRNAs) [28]. In the nucleus, m^1^A modifications are catalyzed by the methyltransferase complex TRMT6/61A and reversed by the demethylases ALKBH1 and ALKBH3. In mitochondria, m^1^A modification on mt‐tRNA and a subset of mt‐mRNA are catalyzed by TRMT61B and TRMT10C [27, 29]. Notably, m^6^A mark on mRNA is six times more prevalent than m^1^A [30].

### 2.2 Cytosine Methylation (m^5^C, 5hmC, m^3^C)

Methylation at the *N* ^5^ position of cytosine on RNA molecules, known as m^5^C, is present at significantly lower abundance than m^6^A modifications [31]. These m^5^C sites are predominantly observed within mRNAs, lncRNA species, and small regulatory ncRNAs such as tRNAs [32, 33, 34]. Notably, m^5^C sites exhibit a significant enrichment in the untranslated region (UTRs) at both the 5' and 3' ends of mRNA, as well as near the translation initiation codon, indicating its role in translation and RNA stability [35]. Besides, m^5^C sites are found in rRNA. During the maturation of ribonucleoprotein complexes, rRNAs undergo various chemical modifications, including m^5^C modifications in the 28S/25S rRNA mediated by methyltransferase NSUN5 and NSUN1 [36, 37]. These m^5^C modifications augment base stacking through increased thermal stability of hydrogen bonding with guanine, thereby facilitating stabilization of rRNA folding within functionally crucial ribosomal regions [38, 39]. Interestingly, m^5^C on chromatin‐associated RNA can be oxidized by ten–eleven translocation (TET)‐family enzymes to 5‐hydroxymethylcytosine (5hmC), which could be identified by a specific reader RBFOX2 that degraded the 5hmC‐modified RNAs [40, 41]. However, the role of 5hmC in mammals requires further investigation.

Additionally, the covalent addition of a methyl group to the nitrogen atom at Position 3 of cytosine, known as 3‐methylcytosine (m^3^C), has recently been identified on tRNAs and mRNAs [4, 42]. METTL 2 and 6 catalyzes m^3^C on tRNAs, and METTL8 catalyzes m^3^C on mRNAs [43, 44].

### 2.3 Guanosine Methylation (m^7^G)

m^7^G modification, a highly conserved and prevalent RNA modification, was initially believed to be present exclusively at the 5' caps of eukaryotic mRNA to protect transcripts against exonucleolytic degradation [45]. Recent studies have revealed the presence of m^7^G at various internal positions across multiple types of RNAs [46, 47, 48]. This modification plays a crucial role in diverse aspects of RNA metabolism including processing, stabilization, maturation, and translation [48, 49]. In mammals, m^7^G modification at Position 46 of variable loop at tRNA stabilizes the tertiary structure of tRNA [50]. m^7^G at a G_1639_ location in 18S rRNA promotes maturation of 18S rRNA [51]. Furthermore, m^7^G modification in G‐quadruplex structure of pri‐miRNAs transcripts was shown to facilitate miRNA biogenesis [46].

### 2.4 Isomerization of Uridine/Pseudouridylation (Ψ)

Pseudouridine (Ψ) is formed through the isomerization of the uridine base [52]. This highly conserved RNA modification is ubiquitous and present in coding and noncoding RNAs. Thousands of Ψ sites are identified on mRNAs, with a higher enrichment of Ψ on the coding sequence (CDS) and the 3′UTR [5, 53]. Ψ is implicated in multiple steps of mRNA processing and has primarily been investigated within the context of protein translation. For instance, termination codons modified with Ψ (ΨAA, ΨAG, and ΨGA) have been shown to suppress translation termination in yeast, leading to stop codon read through in mRNAs carrying premature termination codons (PTCs) [54].

Pseudouridylation of rRNA reduces ribosome–ligand interactions and translational fidelity [55]. Ψ is mostly found at Position 55 within the TΨC stem‐loop of almost all tRNAs. Additionally, Ψ occurs less frequently at various positions such as Ψ13 and Ψ39, where they contribute to stabilizing the specific structure of tRNAs [39, 56]. Besides, Ψ was found on tRNA‐derived fragments that regulates protein synthesis [57].

In summary, the precise regulation of these diverse RNA modifications relies on a highly coordinated network of enzymatic complexes. While each modification possesses distinct structural properties and regulatory enzymes, they collectively function to fine‐tune RNA structure and stability. Understanding these fundamental regulatory mechanisms provides the basis for exploring their specific functions in RNA metabolism and genome stability, which will be discussed in the following section.

## 3 Molecular Function of RNA Modifications

Building upon the structural diversity of RNA modifications, we now discuss their functions. RNA modifications regulate various aspects of RNA biology, including splicing, nuclear export, stability, degradation, and translation. These functions are often mediated by reader proteins that specifically recognize and bind to modified sites [58]. Depending on their cellular contexts and locations, RNA modifications exhibit distinct functions, such as enhancing translation, stabilizing RNA during stress, or maintaining genome integrity through DNA damage repair and chromatin interactions. By examining these modifications, we aim to uncover their unique contribution to cellular homeostasis and stress adaptation (Table 1).

**TABLE 1:** Signals and functions of RNA modifications.

Gene/location	RNA modification	Regulator	Mechanisms/functions
mRNA 5‘UTR	m^1^A	TRMT61B	m^1^A facilitating translation initiation or promoting elongation factor recruitment
mRNA CDS	m^1^A	TRMT10C	m^1^A through a mechanism involving ribosomal scanning or translation
Stress granules	m^1^A	TRMT6‐TRMT61A	m^1^A in the CDS exerting an effect on translation elongation and potentially diminishing translation efficiency
Stress granules	m^1^A	ALKBH7	Increasing mitochondrial activity
Stress granules	m^6^A	YTHDF1, YTHDF2, YTHDF3, YTHDC1	Protecting RNA and aiding protein synthesis after the stimulus disappears
5'UTR of p27	m^5^C	NSUN2	Restricting gene translation
3'UTRs of p21	m^5^C	NSUN2	Enhancing p21 expression
IL‐17A mRNA	m^5^C	NSUN2, ALYREF	Enhancing the translation efficiency of IL‐17A
tRNA	m^5^C	Dnmt2	Against stress‐induced tRNA cleavage
Mitochondrial	m^3^C	METTL8	Mitochondrial tRNA and translation
Stress granules	m^7^G	QKI7	Modulating mRNA stability and translation during stressful conditions
Stress granules	Ψ	Pus7	Translation
mRNA	Ψ	Pus1, Pus7	Protein synthesis decreasing

### 3.1 RNA Modifications in RNA Metabolism

The effect of RNA modifications on RNA metabolism greatly relies on their reader proteins, which recognize and bind to specific modification and regulate RNA splicing, nuclear export, stability, degradation, and translation [13, 58]. Since numerous reviews have already elucidated the effect of m^6^A modification on RNA metabolism [59, 60, 61, 62], this review will focus on the functions of non‐m^6^A RNA modifications, mainly including m^1^A, m^5^C, m^3^C, m^7^G, and Ψ.

The molecular function of m^1^A appears to depend on its location. Within eukaryotic mRNAs, m^1^A in highly structured or GC‐rich regions of 5'UTRs, was shown to facilitate translation initiation potentially by modifying the predicted secondary structure of the transcripts [3, 63]. Additionally, the positive charge of m^1^A in the CDS of eukaryotic mRNAs and mitochondrial RNA was shown to interfere with translation [30, 63]. Under proteostatic stress (e.g., heat shock), m^1^A modification is increased in mRNAs and promotes stress granules (SGs) formation [64]. The accumulation of m^1^A‐generating methyltransferase TRMT6/61A and m^1^A‐modified transcripts in SGs was shown to protect RNA and aid protein synthesis after the stimulus disappears [65].

Reminiscent of the role of m^6^A reader proteins, specifically the YTH domain‐containing family proteins, in accumulating m^6^A in SGs [66, 67, 68, 69], it is conceivable that such reader proteins may also be involved in granulation and safeguarding of m^1^A‐contained transcripts under heat shock stress. In vitro data suggests that m^6^A reader proteins, including YTHDF1, YTHDF2, YTHDF3, and YTHDC1, can bind to m^1^A on mRNA [70]. Hence, further discussion on the interaction between m^1^A and YTH domain‐containing family protein in cellular SGs is necessary.

Recent studies on m^5^C mRNA modification indicate that m^5^C regulates transcript export and translation efficiency [71, 72]. Deletion of the m^5^C methyltransferase *NSUN2* in human diploid fibroblasts (HDFs) leads to increased levels of p27, and m^5^C formation in the 5'UTR of p27 can restrict gene translation [73]. The m^5^C modification in mRNA coding regions significantly reduces translation in bacterial and HeLa cell extracts [74, 75]. Interestingly, m^5^C modifications at the 3'UTRs of *p21* mRNA coordinate with m^6^A modifications to enhance *p21* expression [74]. Moreover, the addition of m^5^C modification at *Interleukin‐17a* (*Il‐17a*) mRNA has been demonstrated to enhance the translation efficiency of IL‐17A [76]. ALYREF, the m^5^C reader, directly binds to m^5^C in mRNA through a unique RNA‐binding motif and mediates the mRNA export from the nucleus [77]. As for m^5^C modification on tRNAs, both the methyltransferase NSUN2 and DNMT2 have been shown to protect tRNA from stress‐induced tRNA cleavage [78, 79]. Besides, high‐throughput analysis revealed that 5hmC is predominantly located within introns and exons of coding transcripts through the action of Tet‐family enzymes [40, 80]. The association of 5hmC with translation activation in drosophila is different from m^5^C modification [81].

Recent studies on m^3^C RNA modification indicate its regulatory role in mitochondrial tRNA and translation, with METTL8 identified as the enzyme catalyzing m^3^C formation [82, 83, 84]. METTL8 exists in two isoforms with distinct cellular localizations: METTL8‐iso1 targets mitochondria via an N‐terminal pre‐sequence, while METTL8‐iso4 predominantly resides in the nucleolus, indicating diverse functions in these compartments. Study in *Escherichia coli* have demonstrated the importance of Positions 32 within tRNA anticodon loop for ribosome binding and translation fidelity, suggesting that m^3^C contributes to translation regulation [85, 86]. Although *METTL8* depletion in mitochondria does not affect Hsmt‐tRNAThr/Ser (UCN) aminoacylation or ribosome association, it alters the synthesis of mitochondrial‐encoded proteins [83, 87]. Although the molecular mechanism underlying how m^3^C_32_ affects translation remains unclear, it has been indicated that m^3^C_32_ may interact with Positions 32 and 38 of the anticodon loop to maintain an optimal tRNA conformation necessary for its function [88, 89].

m^7^G RNA modification plays a regulatory role in translation efficiency [90, 91]. In tRNA, m^7^G modification is located at Position 46 of tRNA variable loop and is catalyzed by METTL1 and WDR4 heterodimeric complex [92]. m^7^G modification was found to protect tRNA from rapid tRNA decay [93]. Recent studies further reveal that this modification regulates the biogenesis of tRNA‐derived small RNAs (tsRNAs) [94]. In mRNA, the biological functions of m^7^G cap modifications have been extensively reviewed, and herein we specifically focus on the internal m^7^G marks [95]. Similar to m^1^A, internal m^7^G modifications possess a positive charge, enabling them to modulate protein–RNA interactions and reorganize local RNA secondary structures, thereby influencing their biological functions. METTL1 catalyzes internal m^7^G modifications, which have been shown to enhance mRNA translation efficiency [48]. Notably, QKI7, identified as an mRNA internal m^7^G‐binding protein, interacts with the SG core protein G3BP1 via its C terminus. This interaction facilitates the sequestration of m^7^G‐modified transcripts into SGs, thereby modulating mRNA stability and translation during cellular stress [96]. Besides, IGF2BP family proteins IGF2BP1‐3 could bind to internal mRNA m^7^G and promotes mRNA degradation [97]. However, further study is needed to uncover other methylases, readers, and demethylases involved in the regulation of internal m^7^G modifications.

Recent studies highlight the role of Ψ in pre‐mRNAs splicing, mRNA stability, and translation efficiency [98]. Ψ modification, located near the 3’ splice site within the polypyrimidine tract and regulated by U2 auxiliary factor (U2AF), have been shown to impede pre‐mRNA splicing [99]. A more recent study revealed that Ψ occur cotranscriptionally in pre‐mRNAs, enriched near splice sites and RNA‐binding protein binding regions, in which they regulate mRNA alternative splicing and 3′ end processing through the actions of tissue‐specific pseudouridine synthases including PUS1, PUS7, and RPUSD4. As for mature mRNA, the stability and translation are regulated by Ψ. For example, heat shock‐induced, Pus7‐dependent pseudouridylation increases translation efficiency by approximately 25% compared to *Pus7* knockdown in yeast [100]. However, another study showed that Ψ‐containing mRNA exhibit a 30% reduction in protein expression, and a Ψ modification at the third codon position of “UUU” specifically limits bacterial mRNA translation [75, 101]. Furthermore, both in vitro and in vivo studies have shown that Ψ modifications can convert nonsense codons into sense codons [54, 102]. Based on these findings, targeted pseudouridylation of mRNA can suppress nonsense‐mediated mRNA decay and promote read through of PTCs, offering a gene‐specific approach for nonsense suppression in human cells [103].

However, some of these findings were derived from experiments with artificial mRNA, and further research is needed to elucidate the function of Ψ in cellular contexts.

### 3.2 RNA Modifications in Genome Integrity

**FIGURE 2:**
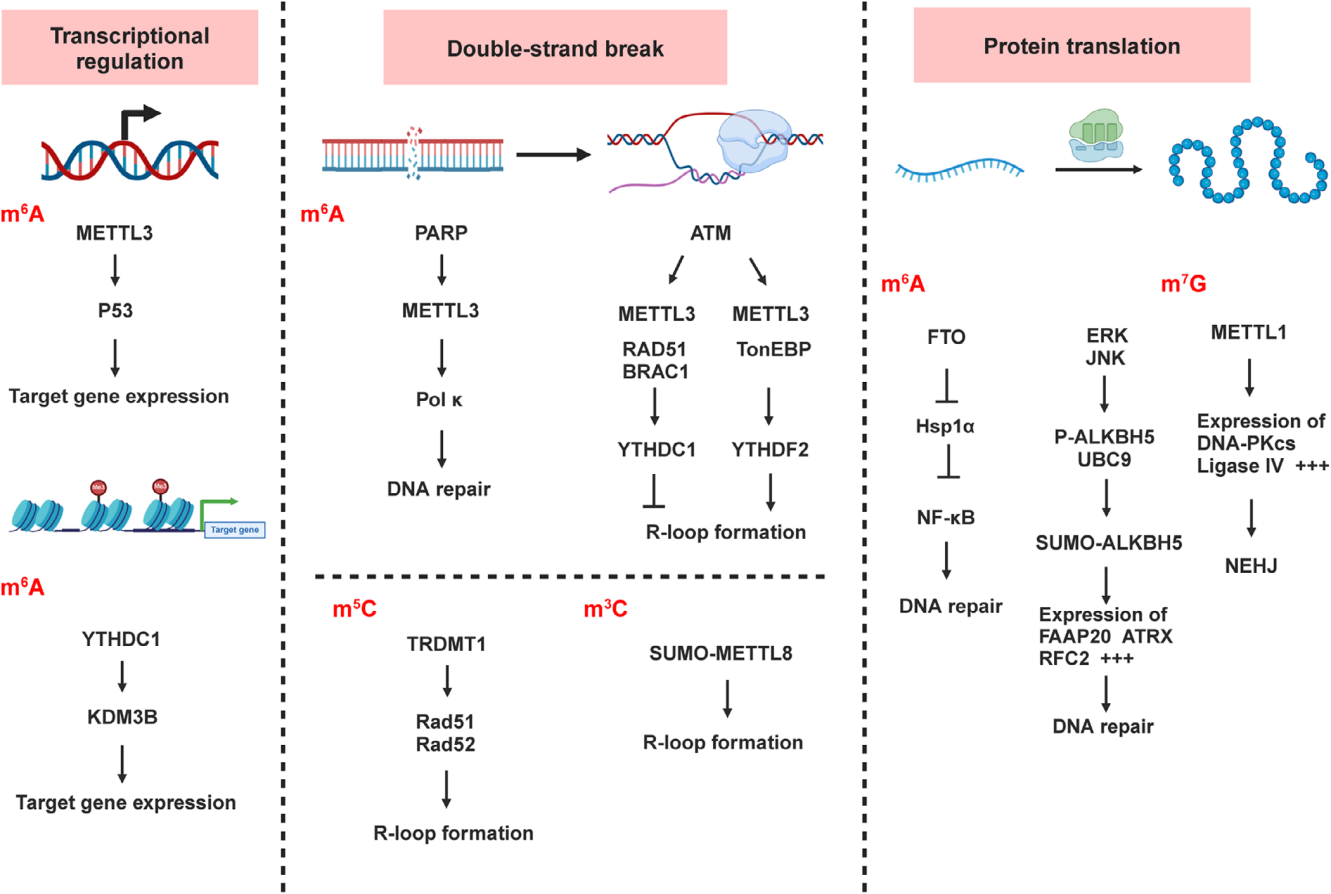
As our understanding of the intricate interplay between RNA modifications and transcript properties deepens, we are gaining profound insights into the pivotal roles these subtle chemical alterations play in metabolic processes and their impact on physiology. In cellular metabolism, distinct pathways may converge onto a common signaling pathway with disparate functions. In recent years, corroborating reports have emerged to confirm the crucial role of RNA modifications in maintaining genome integrity through diverse mechanisms. This image was created with BioRender (https://biorender.com/).

In recent years, studies have confirmed that m^6^A RNA modification plays critical roles in maintaining genome integrity through its involvement in DNA damage repair, chromatin structure, and genomic stability (Figure 2) [104, 105, 106].

As for DNA damage, m^6^A‐modified RNAs are dynamically recruited to DNA damage sites and play a role in facilitating repair processes. For instance, after UV‐irradiated DNA damage, RNA m^6^A modification is rapidly and transiently induced. In this process, METTL3 enables the rapid recruitment of DNA polymerase κ (Pol κ) to damaged chromatin regions, thereby promoting efficient nucleotide excision repair, while METTL3 deficiency delays repair and increases UV sensitivity [107]. Additionally, ataxia telangiectasia mutated kinase (ATM) promotes m^6^A deposition on RNAs associated with double‐strand break (DSB) repair through METTL3 activation [105]. Apart from METTL3, YTHDC1 functions as a key regulator of DDR, orchestrating both m^6^A‐dependent intron retention and m^6^A‐independent *TP53* transcriptional pause‐release, thereby maintaining genomic stability and preventing aberrant cancer cell proliferation [108].

Conversely, the m^6^A demethylase FTO has also been found to protect cells from DNA damage by enhancing the stability of transcripts of DNA repair genes, such as *Hspa1a*, through the removal of m^6^A modifications in mouse osteoblasts [109]. These findings suggest m^6^A methylases (e.g., METTL3) and demethylases (e.g., FTO) dynamically regulate DDR protein levels by modulating their translation processes.

The interaction between m^6^A and chromatin plays a crucial role in maintaining genomic stability [110, 111, 112, 113]. It has been reported that upon micro‐irradiation, m^6^A RNA translocate from the cytoplasm to the nucleus. Mechanistically, ROS activate the ERK/JNK signaling pathway, driving the phosphorylation of m^6^A demethylase ALKBH5 at Ser87 and Ser32. Subsequently, the phosphorylated ALKBH5 interacts with UBC9, a SUMO E2 conjugating enzyme, resulting in the SUMOylation of ALKBH5 at Lys86 and Lys321. These posttranslational modifications inhibit the ALKBH5 activity, leading to elevated m^6^A signals on DNA repair gene transcripts and enhanced cellular protection against ROS‐induced damage [114]. On the other hand, deletion of *Suv39h*1/h2 leads to the loss of H3K9me3 histone marks, resulting in DNA hypomethylation and genomic instability, which reduced accumulation of m^6^A‐modified RNA at micro‐irradiation–induced damage sites [115]. Furthermore, m^6^A modification interacts with the transcription factor p53, modulating its damage‐responsive transcriptomic activity and further linking chromatin dynamics with the DDR [116].

m^6^A exerts dual regulatory functions in the formation and resolution of R‐loop, which is essential for genomic stability [117, 118]. The METTL3/METTL14 complex mediates m^6^A deposition on nascent RNA, stabilizing R‐loops near transcription termination sites to facilitate efficient transcription termination [119]. Following study identified the RNA helicase DDX21 as a key factor in directing co‐transcriptional m^6^A modification by interacting with METTL3 and recognizing R‐loops [118]. At sites of DNA damage, METTL3 is recruited to R‐loops, where increased m^6^A modification recruits the reader protein YTHDC1. Then, YTHDC1 promotes homologous recombination (HR) by enhancing R‐loop accumulation at DSBs [120]. Conversely, aberrant and excessive R‐loop accumulation poses a risk to genomic stability. Abakir et al. showed that the m^6^A reader protein YTHDF2 mitigates this by clearing R‐loops within repetitive elements and intronic regions in human pluripotent stem cells, thereby preserving genomic homeostasis [121]. Another study also revealed that ARID1A recruits METTL3 and METTL14 to R‐loops, where m^6^A modification facilitates RNase H1 recruitment, which promotes R‐loop resolution and DNA end resection to maintain genome stability [117]. The dual functions of m^6^A in R‐loop regulation are attributed to interaction with distinct reader proteins, such as YTHDC1 and YTHDF2, whose recruitment may be regulated by specific factors. Therefore, further investigation is needed to elucidate these mechanisms. Importantly, these findings highlight the complex and sometimes opposing roles of m^6^A modifications in DNA repair. The controversy in current literature can be partially explained by distinguishing between local and global effects. On one hand, m^6^A deposition acts as a positive signal at specific DNA damage sites, where it recruits reader proteins like YTHDC1 to facilitate repair factor assembly. On the other hand, m^6^A modification on the mRNAs of DNA repair genes often promotes their degradation via YTHDF, thereby reducing the level of repair proteins. Thus, the overall impact of m^6^A on genome integrity is determined by the balance between its direct role in chromatin remodeling and its indirect regulation of repair gene stability.

Apart from m^6^A, other RNA modifications contribute to DNA damage signaling. Recent study suggests RAD52 as a potential reader protein for m^5^C in DNA:RNA hybrids during DNA repair [122]. Besides, Fragile X mental retardation protein (FMRP) is identified as an m^5^C reader. FMRP coordinates the m^5^C writer TRDMT1 and eraser TET1 function to promote transcription‐coupled HR and R‐loop resolution, thereby facilitating DSB repair [123]. SUMOylated METTL8 have been shown to add m^3^C modification, which stabilizes R‐loops at selected gene regions. Knockout of *Mettl8* reduces R‐loops and enhances genomic stability [124]. Moreover, METTL1‐mediated m^7^G tRNA modification selectively enhances the translation of DNA repair proteins, such as DNA‐dependent protein kinase catalytic subunit or DNA ligase IV, following ionizing radiation (IR). This leads to an enhanced efficiency of nonhomologous end‐joining (NHEJ)‐mediated DSB repair [125].

Taken together, current studies indicate that RNA modifications are dynamic regulators of RNA lifecycle and genome stability. Through their involvement in RNA metabolism and DNA damage repair, these modifications support cell survival under diverse stress conditions. These molecular functions are particularly important in the context of cancer, where they contribute to the survival and functional regulation of both tumor and stromal cells within the TME.

## 4 RNA Modifications in TME

**FIGURE 3:**
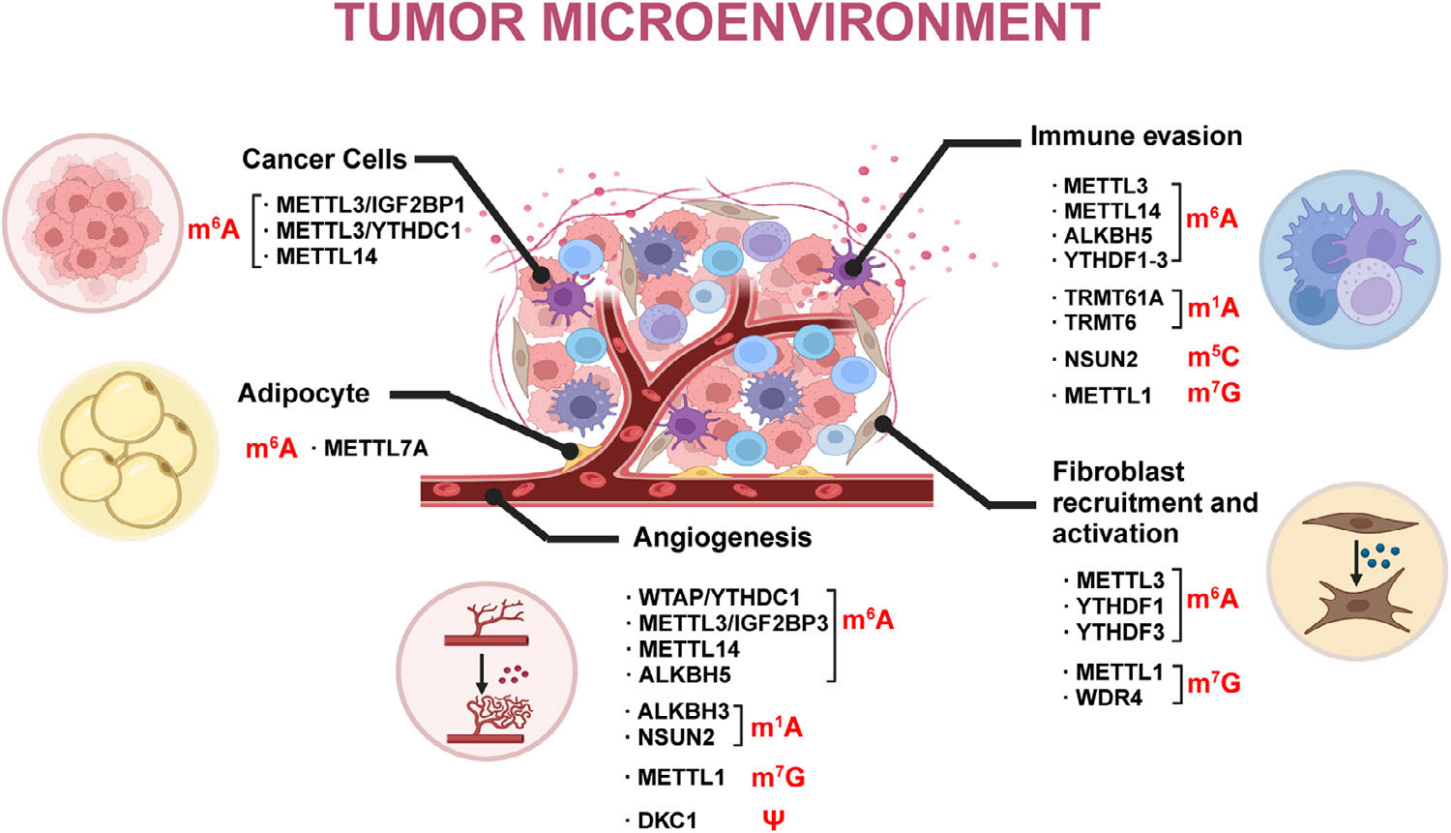
Tumor initiation and progression necessitate a supportive microenvironment; however, the components of the tumor microenvironment (TME) exhibit substantial heterogeneity rather than uniformity. These components are influenced by signaling pathways, cytokines, and RNA modification as a crucial factor. This dynamic process facilitates interactions between cancer cells and noncancerous cells within the TME, enabling cancer cells to evade destruction and progress into fully developed tumors. This image was created with BioRender (https://biorender.com/).

During tumorigenesis, malignant cells release signals that trigger an immune response. While an effective early immune influx can eliminate these cells and prevent tumor formation, inadequate signaling or insufficient immune activity may allow tumors to emerge, grow locally, and eventually metastasize. This progression occurs within the TME, which represents a highly organized niche comprising cancer cells surrounded by diverse nonmalignant cell types [126]. The noncancerous cellular components encompass various immune cells, CAFs, ECs, pericytes, and tissue‐specific cell types such as adipocytes and neurons. As the tumor progresses, the TME undergoes dramatic structural and compositional changes, modulating the interactions between tumor and stromal cells. While the cellular components of the TME are well characterized, the molecular mechanism governing their crosstalk remains largely unknown. In this section, we explore how RNA modifications act as critical orchestrators of these intercellular communications, modulating processes from immune evasion to vascular remodeling, and impact tumor proliferation and metastasis (Figure 3).

### 4.1 RNA Modifications in Immune System

Extensive evidence has demonstrated the intricate involvement of RNA modifications in immune system, which includes innate and adaptive immune response [127]. Immunotherapy, specifically the programmed cell death protein 1 (PD‐1)/programmed cell death ligand 1 (PD‐L1) signaling‐mediated immune checkpoint, has shown great therapeutic potential against tumor, in which RNA modification plays a crucial role [128, 129].

#### 4.1.1 RNA Modifications in Innate Immune System

The innate immune system of the TME mainly comprises macrophages, neutrophils, natural killer (NK) cells, and dendritic cells (DCs). The phenotype of them is highly plastic, and depending on the diverse signals from the tumor, these cells play an either antitumor or protumor role.

Tumor‐associated macrophages (TAM) are abundant in TME and are often reprogrammed into functional heterogeneous populations, such as activated tumor‐opposing M1 and activated tumor‐supporting M2 macrophages [130, 131]. Reprogramed TAM are the major drivers of T cell exhaustion. Recent studies have revealed the role of m^6^A modification in regulating TAM function. A subcluster of C1q^+^ TAM was identified that exhibited a unique RNA m^6^A methylation phenotype and regulated tumor‐infiltrating CD8^+^ T cells. Deficiency of m^6^A methyltransferase *Mettl14* or m^6^A reader protein *Ythdf2* in mice macrophages upregulated the level of cytokine subunit *Ebi3* transcripts, which impeded the antitumor function of CD8^+^ T cells and promoted tumor growth [132]. Therefore, the m^6^A modification on *Ebi3* is a switch determining the immunosuppressive phenotype of TME. Furthermore, METTL3‐mediated m^6^A modification enhances the transcriptional stability and expression of *STAT1* mRNA, which promotes M1 polarization and improves the response to anti‐PD‐1 therapy in colorectal cancer (CRC) and melanoma [133]. Conversely, another study showed that deletion of *Mettl3* or the m^6^A reader *Ythdf1* in TAM led to augmentation of M1/M2‐like TAM and regulatory T (Treg) cell infiltration, thereby promoting tumor growth and metastasis. Mechanistically, METTL3 mediated m^6^A modification on the *Spred2* transcript. Deletion of *Mettl3* hampered YTHDF1‐mediated translation of SPRED2, thereby activating NF‐κB and STAT3 signaling [134]. Besides, it has recently been found that H3K18 lactylation upregulates METTL3‐mediated m^6^A modification of *Jak1* mRNA in myeloid cells. The m^6^A‐YTHDF1 axis enhances *JAK1* translation and subsequent STAT3 activation, thereby driving the immunosuppressive functions of myeloid cells [135].

In addition to cytokine secretion, macrophages can communicate with tumor cells via exosomes. Recent evidence highlights RNA modifications as a key regulator of this process. The RNA‐binding protein HNRNPA2B1 has been identified to specifically bind and sort m^6^A‐modified miRNAs into exosomes [136, 137]. This sorting mechanism significantly impacts tumor progression. For example, in glioma and triple‐negative breast cancer (TNBC), HNRNPA2B1 sorts specific noncoding RNAs into exosomes, which are then transmitted to macrophages to induce immunosuppressive polarization and promote tumor progression [138, 139]. Besides, TAM‐derived exosomes regulate the m^6^A landscape in medulloblastoma cells by downregulating METTL14, which upregulates the target gene *FOXD1* to promote tumor progression and immune evasion. Furthermore, METTL3‐mediated m^6^A modification of *RAB27A* mRNA enhances exosome biogenesis and secretion, which shifted the peritoneal macrophages to establish a pre‐metastatic niche in gastric cancer [140]. Therefore, RNA modification plays an active role in the immune‐modulatory function of TAM and suggests potential therapeutic strategies of targeting m^6^A in macrophages to enhance tumor immunotherapy.

DCs, a type of antigen‐presenting cells (APCs) involved in the immune response, serve as a crucial link between innate and adaptive immunity [141]. DCs play a pivotal role in T cell interactions within both tissue lymph nodes and the TME [142]. Recently, it has been reported that RNA m^6^A modification plays a critical role in regulating the antitumor immune effects of DCs. m^6^A signals were identified on transcripts relating to lysosomal proteases, which were read and bound by YTHDF1 that promotes their translation. Depletion of *Ythdf1* in DCs enhances the cross‐presentation of tumor antigens and promotes the cross‐activation of CD8^+^ T cells in vivo, which exerts antitumor effect [143]. Complete abrogation of antitumor response is observed in *Ythdf1–* ^/–^ mice simultaneously lacking CD8^+^ T cells. More importantly, the therapeutic outcome of PD‐L1 immunotherapy was greatly enhanced in *Ythdf1*‐knockout mice [143]. A more recent study also reveals that YTHDF1 mediates resistance to radiotherapy by degrading STING and reducing IFN‐I production in DCs in a murine melanoma model [144]. Therefore, these findings suggest that targeting to YTHDF1 in DCs is a potential strategy for immunotherapy.

NK cells are cytotoxic lymphocytes of the innate immune system, characterized by their ability to produce cytokines and kill target cells [145]. They play a crucial role in viral and intracellular bacterial infections, as well as in tumor immunosurveillance [146, 147]. It is well established that the, development, homeostasis, and antitumor effect of NK cells are strengthened by m^6^A modification [148]. For instance, METTL3‐mediated m^6^A ensures an appropriate response of NK cells to IL‐15 stimulus by promoting *SHP‐2* translation [149]. Mice with conditional deletion of *Mettl3* in NK cells exhibited compromised infiltration and impaired tumor immunosurveillance within the TME, thereby promoting tumor progression and reducing mouse survival rates [149]. On the other hand, m^6^A eraser FTO negatively regulates NK cell immunity by enhancing the stability of *suppressor of cytokine signaling protein (SOCS)* family mRNAs. Therefore, FTO deficiency promotes NK cell activation and consequently improves antitumor responses in melanoma and leukemia [150]. A recent report also indicated that YTHDF2 positively regulates the antitumor and antiviral activity of NK cells. YTHDF2 is required for the homeostasis, terminal maturation, and effector function of NK cell [151]. Overall, these observations have demonstrated the involvement of m^6^A modification in innate immunity and implicated the therapeutic potential of targeting to RNA modification to promote immunotherapy.

#### 4.1.2 RNA Modifications in Adaptive Immune System

The adaptive immune system comprises T and B lymphocytes that rapidly respond to antigens presented by APCs and generates both rapid defense and long‐lasting memory [152]. The activation of T cells is crucial in adaptive immune responses, particularly CD4^+^ and CD8^+^ T cells in antitumor immunity, which is greatly regulated by RNA modifications [153]. Depletion of *Mettl3* in CD4^+^ T cells disrupted T cell homeostasis and differentiation by downregulating IL‐7‐mediated STAT5/ SOCS activation [154]. Additionally, conditional deletion of *Mettl3* in CD4^+^ T cells diminished T follicular helper (Tfh) cell differentiation and functional maturation, subsequently inhibiting the antibody response of B cells due to impaired stability of m^6^A‐modified *Tcf7* [155]. Another study further shows that YTHDF2 supports Treg cells function within TME by accelerating the degradation of m^6^A‐modified transcripts encoding NF‐κB negative regulators. Loss of YTHDF2 in Tregs impairs their suppressive function and promotes tumor regression [156]. However, it is reported that m^6^A modification supported the Treg suppressive effect on CD8^+^ T cells [157]. Therefore, selective manipulation of m^6^A modification in different cells are crucial to cancer immunotherapy.

RNA modifications of the tumor cells also help them establish immune evasion by modulating T cells within TME. For instance, METTL3 in CRC cells activated the m^6^A‐BHLHE41‐CXCL1/CXCR2 axis to recruit myeloid‐derived suppressor cells, which inhibited the activation and proliferation of CD4^+^ and CD8^+^ T cells, ultimately promoting CRC progression [158]. METTL3‐ or METTL14‐deficient tumors enhanced IFN‐γ‐Stat1‐Irf1 signaling by stabilizing the mRNA of *Stat1* and *Irf1* through YTHDF2, thereby augmenting the infiltration of cytotoxic CD8^+^ T cells into TME and increasing secretion of IFN‐γ, CXCL9, and CXCL10 in vivo [133]. On the other hand, tumor cells lacking m^6^A demethylase ALKBH5 exhibited enhanced sensitivity to cancer immunotherapy. ALKBH5 exerted regulatory effects on m^6^A density and splicing events within tumors during immune checkpoint blockade (ICB). Additionally, ALKBH5 modulated the expression of Mct4/Slc16a3 and lactate content in the TME, as well as influenced the composition of tumor‐infiltrating Treg and myeloid‐derived suppressor cells [159].

#### 4.1.3 RNA Modifications in PD‐L1 Response

The interaction of programmed cell death 1 ligand 1 (PD‐L1) interaction with its receptor PD‐1 inhibits T cell activation [160]. Many tumors express PD‐1 ligands, which bind to PD‐1 on T cells patrolling the TME and exerts immunosuppressive effect. Therefore, ICB therapy targeting to PD‐1/PD‐L1 axis has been developed and approved for treatment in different solid tumors.

The efficacy of PD‐1/PD‐L1 therapy is modulated by RNA m^6^A modifications, which regulate tumor immune evasion through the expression of PD‐L1 in tumor cells and PD‐1 in T cells. For example, in non‐small‐cell lung carcinoma cells, METTL3 facilitates the m^6^A modification of *circIGF2BP3* and promotes its circularization through a YTHDC1‐dependent mechanism. This process establishes a regulatory axis involving circIGF2BP3, PKP3, and OTUB1, which increases PD‐L1 levels by deubiquitination, ultimately promoting immune evasion [161]. Interestingly, in hepatocellular carcinoma (HCC), WTAP‐mediated m^6^A modification enhances the stability of *circCCAR1* via IGF2BP3. Secreted circCCAR1 can be internalized by CD8^+^ T cells, leading to their dysfunction through the stabilization of the PD‐1 protein. This mechanism drives resistance to anti‐PD‐1 immunotherapy, suggesting a potential therapeutic strategy for HCC patients [162]. The m^6^A demethylase ALKBH5 regulates MAP3K8 expression in an m^6^A‐dependent manner, promoting HCC cell proliferation and metastasis. Additionally, ALKBH5 increases IL‐8 expression, facilitating the recruitment of PD‐1^+^ macrophages and contributing to tumor progression [163]. In bladder cancer cells, m^6^A modification present on *PD‐L1*, recognized by the m^6^A reader IGF2BP1, enhances RNA stability and PD‐L1 expression. This mechanism, correlated with activated JNK signaling, facilitates immune evasion by suppressing CD8+ T cell cytotoxicity [164].

Apart from m^6^A, other modification, such as tumor‐intrinsic m^7^G tRNA modification by METTL1, enhance the translation of *CXCL8* in humans and *CXCL5* in mice. This process induces resistance to anti‐PD‐1 therapy through inhibition of CD4^+^ T‐cell expansion and suppressing the antitumor activity mediated by both CD4^+^ and CD8^+^ T cells [165].

Collectively, these studies demonstrate that the function of RNA modifications in the immune microenvironment is highly context dependent. The same regulator can exert opposite effects depending on the specific cell type. An example is the reader protein YTHDF2. In Treg cells, it promotes tumor progression by maintaining their suppressive function, whereas in NK cells, it inhibits tumor growth by supporting effector activity and homeostasis [151, 156]. These distinct outcomes are likely driven by the specific set of mRNA targets available in different cells. Furthermore, microenvironmental factors, such as local cytokine levels and metabolic conditions, may differentially regulate the expression and activity of these RNA‐modifying enzymes, thereby dictating whether they function as tumor promoters or suppressors in different context.

### 4.2 RNA Modifications in ECM

CAFs represent the predominant stromal cell population within the TME, and contribute significantly to ECM synthesis and remodeling [166, 167]. ECM serves as a noncellular structural component within TME, functioning as a reservoir for secreted molecules and as a substrate for cell adhesion and migration. This dynamic interplay facilitates effective intercellular communication within TME [168].

METTL3‐mediated m^6^A modification increases the expression of COL10A1 in CAFs, which promotes the proliferation of lung squamous cell carcinoma (LUSC) cells and inhibits apoptosis‐induced oxidative stress [169]. Another study showed that METTL3 increase the level of m^6^A modification on *RAC3* in CAFs, promoting migration and invasion of non‐small cell lung cancer (NSCLC) cells [170]. In hepatic stellate cells (HSC), the substantial production of ECM is attributed to the enhancement of mRNA stability in major cluster of collagen genes through YTHDF1‐mediated m^6^A modification, thereby offering novel targets for epigenetic therapy in liver fibrosis [171].

METTL1 and WDR4 are highly expressed in osteosarcoma and are correlated with unfavorable prognosis. METTL1/WDR4‐mediated m^7^G modification of tRNAs enhances the translation efficiency of mRNAs with a higher frequency of m^7^G tRNA‐decoded codons, including ECM remodeling effectors, thereby promoting osteosarcoma progression, and conferring resistance to doxorubicin treatment [172]. The potential role of other RNA modification on CAF function remains unexplored.

### 4.3 RNA Modifications in Vascular Supply

Angiogenesis plays a critical role in tumor development and progression. Tumors growing beyond 1–2 mm require their own vascular network to supply oxygen and nutrients [173]. The absence of angiogenesis is believed to be a key reason why some microscopic lesions remain dormant and do not progress to invasive cancer [174]. Antiangiogenic therapies have two distinct, mutually exclusive effects: They disrupt tumor blood vessels, creating a hypoxic TME that limits nutrient availability and slows tumor growth. However, compensatory mechanisms involving alternative angiogenic pathways often lead to resistance to vascular endothelial growth factor (VEGF) or VEGF receptor (VEGFR) inhibition, reducing the clinical efficacy of these therapies [175].

RNA modifications, particularly m^6^A methylation, play a critical role in regulating angiogenesis by stabilizing key angiogenic mRNAs such as VEGFA and heparin‐binding growth factor (HDGF), thereby promoting tumor progression [176, 177, 178]. METTL3, for example, increases the m^6^A levels of *EphA2* and *VEGFA*, which are stabilized by IGF2BP2/3 to drive vasculogenic mimicry (VM) and tumor development in CRC [179]. Additionally, the WTAP/YTHDC1/VEGFA axis has been identified as a potential biomarker for CRC due to its pivotal role in angiogenesis [180]. In gastric cancer, METTL3 upregulates the m^6^A levels of IGF2BP3‐stabilized HDGF, promoting angiogenesis and liver metastasis [181]. Similarly, in bladder cancer, METTL3 enhances the TEK/PI3K/VEGF cascade to stimulate angiogenesis [182]. Besides METTL3, METTL14 plays a crucial role in breast cancer progression and metastasis by regulating the m^6^A on angiogenesis‐ and EMT‐related genes, including *TGFβ*, *SMAD3*, *VEGFA*, and *HMGA2*. This regulation also involves controlling the expression of the m^6^A demethylase *ALKBH5*, which further influences mRNA stability and translation in an m^6^A‐dependent manner, highlighting their combined contributions to tumor development [183]. Moreover, ALKBH5 stabilizes *PVT1* mRNA and promotes angiogenesis in lung cancer [184]. FTO is found to demethylate *VEGFA* and *EGR1* m^6^A modification, which enhances their stability by inhibiting the YTHDF2‐mediated mRNA decay pathway [185]. These findings highlight m^6^A as a key regulator of angiogenesis across multiple cancer types and suggest their therapeutic potential in inhibiting tumor growth and metastasis.

Beyond m^6^A, other RNA modifications, including m^1^A, m^5^C, m^7^G, and Ψ, have been shown as potential regulators of angiogenesis, although their roles remain less explored. The m^1^A demethylase ALKBH3 has been shown to regulate VEGF expression and affect cancer angiogenesis, yet the exact role of m^1^A in this process remains unclear [186]. Similarly, NSUN2‐mediated m^5^C modification plays a role in invasion, metastasis, and angiogenesis in HCC, but its molecular dynamics and specific function in angiogenesis require further investigation [34]. METTL1 upregulates m^7^G methylation of *VEGFA* and enhances its translation in ischemic conditions, facilitating postischemic angiogenesis. While this study is not tumor‐specific, it suggests a potential role for METTL1 in tumor angiogenesis [187]. Additionally, a decrease of Ψ modification in 28S rRNA has been linked to the dysregulation of crucial mRNA translation processes, including those involving VEGF and p53 [188]. The upregulation of DKC1, the enzyme catalyzing Ψ, and its impact on rRNA Ψ in angiogenesis remains unclear, and the role of Ψ in TME across cancers is largely unexplored.

### 4.4 RNA Modifications in Adaptation to Hypoxia

As the rapid and uncontrolled proliferation of tumors limits the availability of oxygen, hypoxia becomes a hallmark of nearly all solid tumors [189]. The presence of hypoxic regions serves as an independent prognostic factor for cancer. Tumor cells, while adapting to hypoxia, exhibit more aggressive and therapeutically resistant tumor phenotypes [190]. It is found that the m^6^A‐modified mRNA binding protein YTHDF1 participate in the hypoxia adaptation and promotes NSCLC progression [191]. Besides, hypoxia induces HIF‐dependent expression of the m^6^A demethylase ALKBH5, which demethylates the 3′‐UTR of *NANOG* mRNA, thereby promoting the specification of breast cancer stem cells [192]. In conclusion, understanding hypoxic adaptation mechanisms is crucial for advancing m^6^A‐based tumor immunotherapies, with future strategies focusing on integrated approaches targeting to m^6^A, hypoxia, metabolism, and immune interactions to improve clinical outcomes.

### 4.5 RNA Modifications in Cancer‐Associated Adipocytes

Many epithelial tumors grow near or metastasize to adipose tissue. As tumors develop, crosstalk between adipose tissue and cancer cells reprograms the complex paracrine and autocrine communication that support tumor growth, with RNA modifications playing a significant role. The adipocyte‐rich TME contributes to cancer therapeutic resistance through intricate mechanisms. For example, in multiple myeloma (MM), methyltransferase METTL7A participates in lncRNA m^6^A methylation and regulates the adipocyte exosomal packaging of LncRNAs, contributing to drug resistance [193]. However, apart from m^6^A, studies on other RNA modifications involved in adipocyte reprogramming within the TME are limited. Therefore, the interaction between adipose tissue and cancer cells warrants further investigation.

### 4.6 RNA Modifications Affect Tumor Proliferation and Metastasis via DDR Pathways

RNA modifications play a crucial role in tumor proliferation, metastasis, and the DDR, particularly in the context of chemotherapeutic drug‐induced DDR. Targeting the RNA modification may enhance cancer cell sensitivity to chemotherapeutic drugs. For example, the METTL3/YTHDC1 axis promotes EGF synthesis through m^6^A modification, which enhances HR and cell survival under Adriamycin treatment in breast cancer [194]. Similarly, in CD133^+^ gastric cancer stem cells, METTL3 facilitates oxaliplatin resistance by stabilizing PARP1 mRNA, which increases base excision repair pathway activity [195]. Beyond these mechanisms, METTL3 also stabilizes p53 protein independently of m^6^A modification in response to DNA damage, though the role of RNA in this interaction remains unclear [116].

Other RNA‐modifying enzymes further underscore the complexity of DDR regulation. ZC3H13‐mediated m^6^A modification of *centromeric protein K* (*CENPK*) enhances tumorigenic pathways and DDR, contributing to cisplatin and carboplatin resistance, particularly in cervical cancer [196]. Besides, fisetin has shown potential in amplifying DSBs through ZC3H13‐mediated m^6^A modification on *PHD Finger Protein 10* (*PHF10*), suggesting a novel therapeutic target for pancreatic ductal adenocarcinoma (PDAC) [197]. Meanwhile, the RNA demethylase ALKBH5, regulated by PRMT5, is highly expressed in breast cancer tissues and modulates *breast cancer susceptibility gene* (*BRCA1*) m^6^A modification to regulate doxorubicin sensitivity in breast cancer [198]. Recent research also revealed the important roles of YTHDF family proteins in DDR and chemoresistance. YTHDF1, dependent on METTL14 and mediated by E2F8, promotes S‐phase entry during DNA replication and DDR, positioning it as a tumor promoter and potential therapeutic target in breast cancer. However, the role of E2F8 in DDR remains poorly understood [199]. In addition, the YTHDF2‐E2F3 pathway contributes to chemoresistance in breast cancer by upregulating RAD51 and BRCA1 at both mRNA and protein levels [200]. YTHDF2 also recognizes m^6^A site on *CDKN1B* mRNA and accelerates mRNA decay, with its downregulation increasing DNA damage and sensitizing intrahepatic cholangiocarcinoma (ICC) cells to cisplatin, which may offer insights for therapeutic strategies [201]. Similarly, in TNBC, the RNA‐binding protein POP1 facilitates the YTHDF2‐mediated degradation of m6A‐modified CDKN1A mRNA, thereby promoting tumor proliferation [202]. Additionally, m^5^C RNA modification and R‐loop structures play critical roles in DNA damage repair by promoting transcription‐coupled HR (TC‐HR) and suppressing PARP1‐dependent alternative NHEJ (Alt‐NHEJ). FMRP, as an m^5^C reader, coordinates the activity of m^5^C writer TRDMT1 and eraser TET1 to facilitate m^5^C demethylation and resolve R‐loops, ensuring efficient DSB repair and maintaining genomic stability in cancer cells [123]. These findings underscore the therapeutic potential of targeting m^5^C modifications and associated processes in cancer treatment.

### 4.7 Crosstalk and Coordination Among RNA Modifications

It is important to note that RNA modifications do not work in isolation. Recent evidence suggests that different modifications can cooperate to regulate tumor biology. For instance, m^6^A and m^5^C might function together in the DDR, where the reader protein FMRP binds m^5^C to access chromatin and interacts with m^6^A to facilitate repair [108, 123]. In the context of immune regulation, distinct modifications may contribute to similar outcomes. For example, m^7^G‐mediated translation of CXCL8 and m^6^A‐mediated stabilization of PD‐L1 both contribute to an immunosuppressive microenvironment [161, 165]. These findings indicate that the regulation of the TME involves a combination of multiple RNA modification pathways.

In summary, the findings discussed in this section demonstrate that RNA modifications play a critical role in regulating the diverse components of the TME. By modulating immune cells, stromal components, and vascular networks, these modifications not only facilitate tumor adaptation to metabolic and genotoxic stress but also promote an immunosuppressive environment (Table 2). Given their ability to shape the TME and support tumor survival under stress, targeting specific RNA‐modifying enzymes represents a potential therapeutic approach to inhibit tumor progression.

**TABLE 2:** Cell‐type specific functions of RNA modifications within the tumor microenvironment.

Cell type	Regulator	Molecular target	Mechanism and biological function	Impact on tumor
**Innate immunity**				
**Macrophages (TAMs)**	METTL3	*STAT1*	Increases mRNA stability to promote M1 polarization and antitumor immunity	Inhibit
	METTL3	*SPRED2*	Enhances YTHDF1‐mediated translation to activate NF‐κB/STAT3 signaling	Promote
	METTL3	*JAK1*	H3K18 lactylation‐induced m^6^A promotes JAK1 translation and immunosuppression	Promote
	METTL14/ YTHDF2	*Ebi3*	Loss of m^6^A leads to Ebi3 upregulation, causing CD8+ T cell dysfunction	Promote
	HNRNPA2B1	Exosomal RNAs	Sorts m^6^A‐modified RNAs into exosomes to reprogram TME cells	Promote
**Dendritic cells (DCs)**	YTHDF1	Lysosomal proteases	Promotes translation of cathepsins to limit antigen cross‐presentation	Promote
	YTHDF1	*STING*	Mediates degradation of STING mRNA to impair antitumor immunity	Promote
**Natural killer (NK)**	METTL3	*SHP‐2*	Enhances translation to maintain IL‐15 responsiveness and cell survival	Inhibit
	FTO	*SOCS* family	Demethylation stabilizes SOCS mRNAs to inhibit NK cell activation	Promote
	YTHDF2	—	Essential for NK cell terminal maturation and effector function	Inhibit
**Adaptive immunity**				
**CD4+ T cells**	METTL3	*SOCS* family	Regulates IL‐7/STAT5 signaling to maintain T cell homeostasis	Inhibit
**Tregs**	YTHDF2	*NF‐κB* regulators	Promotes degradation of negative regulators to maintain Treg suppression	Promote
**Stromal cells**				
**CAFs**	METTL3	*COL10A1*	Increases expression to promote cancer cell proliferation and inhibit apoptosis	Promote
	METTL3	*RAC3*	Promotes translation to enhance cancer cell migration and invasion	Promote
	METTL1/WDR4	ECM effectors	m^7^G tRNA modification enhances translation of ECM‐related genes	Promote
**Endothelial cells**	METTL3	*VEGFA*/*EphA2*	Promotes angiogenesis and vasculogenic mimicry via IGF2BP2/3	Promote
	ALKBH5	*PVT1*	Stabilizes lncRNA PVT1 to promote angiogenesis	Promote

## 5 Therapeutic Targeting of RNA Modifications in Cancer

Given the pivotal role of RNA modifications in tumorigenesis and TME regulation, targeting them has emerged as a promising therapeutic strategy [203]. Based on the structural and biochemical information from the catalyzing enzymes, small‐molecule inhibitors are identified. Currently, most of the drugs are in the preclinical exploration phase, with several pioneering agents successfully entering the clinical trials (Table 3).

**TABLE 3:** Small‐molecular drugs targeting RNA modifications.

Target	Compound	Mechanism	Cancer types	Development stage	Key findings/clinical trial ID
METTL3	STC‐15 [204]	Selective catalytic inhibition	Advanced solid tumors	Clinical (Phase I)	First‐in‐class inhibitor; activates innate immunity (NCT05584111)
	STM2457 [205]	Selective catalytic inhibition	AML	Preclinical	Reduces leukemic stem cell growth; predecessor to STC‐15
FTO	Bisantrene (CS1) [206]	Potent FTO inhibition	AML/solid tumors	Clinical (Phase II)	Synergistic with chemotherapy; targets leukemic stem cells (NCT04989335)
	Brequinar [206]	FTO/DHODH inhibition	AML	Clinical (Phase I/II)	No response (NCT03760666)
	FB23‐2 [207]	Direct FTO inhibition	AML, breast cancer	Preclinical	Mimics FTO depletion; suppresses proliferation
	Dac51 [208, 209]	FTO inhibition	T cell acute lymphoblastic leukemia	Preclinical	Potentiates anti‐PD‐L1 blockade; synergizes with T cell immunity
ALKBH5	MV1035 [210]	ALKBH5 inhibition	Glioblastoma	Preclinical	Impairs cell migration and invasion
IGF2BP1	BTYNB [211]	Inhibits RNA binding	Melanoma, ovarian	Preclinical	Blocks binding to c‐Myc/mRNA; reduces metastasis
YTHDFs	Ebselen [212]	YTH domain binder	AML, colon cancer	Preclinical	Disrupts interaction between YTH domain and m^6^A
TRMT6/61A (m^1^A)	Thiram [213]	Complex inhibition	Hepatocellular carcinoma	Preclinical (repurposed)	FDA‐approved drug identified to suppress m^1^A writer complex

The most significant progress made to date involves targeting to m^6^A. STM2457 is a highly selective inhibitor of METLL3, and it has shown therapeutic effects in preclinical models of acute myeloid leukemia (AML) [205]. STC‐15, derived from STM2457, is a first‐in‐class oral inhibitor of METTL3 that entered Phase I clinical trials (NCT05584111, NCT06975293) for patients with advanced malignancies [204]. Preclinical studies demonstrated that STC‐15 not only inhibits tumor growth but also triggers an innate immune response, highlighting its potential to remodel the immune microenvironment [214]. Similarly, targeting the m^6^A demethylase FTO has shown great potential. Bisantrene (CS1), a rediscovered anthracene derivative, was identified as a potent FTO inhibitor and is currently being evaluated in Phase II trials (NCT04989335) for resistant AML [206, 215]. Another dihydoorotate dehydrogenase (DHODH) inhibitor, brequinar, has also been identified to target FTO signaling but showed no treatment efficacy in Phase 1b/2a clinical trial of relapsed or refractory AML (NCT03760666).

Beyond METTL3, inhibitors targeting reader proteins and other modification types are being actively developed. For instance, BTYNB inhibits IGF2BP1‐mRNA binding, showing efficacy in reducing tumor metastasis in preclinical models [211]. Although molecules targeting m^5^C and m^1^A are less studied, early lead compounds like thiram, which blocks TRMT6 and TRMT61A to inhibit m^1^A modification, have shown promise in HCC [213].

Collectively, these developments signify the transition of RNA modifications from biological observations to therapeutic targets. Future efforts will likely focus on improving the specificity of these inhibitors and exploring combinatorial strategies with immunotherapies to overcome drug resistance in the TME.

## 6 Summary and Perspectives

In this review, we overviewed the regulatory mechanisms and functional roles of RNA modifications within the TME. We summarized the enzymatic systems governing adenosine, cytosine, guanosine, and uridine modifications, discussing their fundamental effects on RNA metabolism and genome integrity. Furthermore, we highlighted the specific roles of these modifications in shaping the TME, covering the immune regulation, ECM remodeling, vascular supply, and adaptation to hypoxia. Current evidence indicates that RNA modifications are critical links connecting tumor cell signaling with the stromal microenvironment, ultimately affecting tumor progression and therapeutic response.

While significant progress has been made in RNA modifications, their interplay with the TME remains a great challenge, due to the intricate roles these modifications play in tumorigenesis. Besides, RNA modifications function differently depending on the associated reader proteins, sometimes exerting opposite effects, which requires further study. Future efforts should also focus on expanding research beyond m^6^A modification to underexplored RNA modifications and developing small molecule inhibitors to assess the efficacy of targeting RNA modifications for tumor therapy.

Despite significant progress, deciphering the complexity of the RNA modifications remains a major challenge. Most current studies focus on single modification types, such as m^6^A modification. However, various modifications likely coexist on the same transcript. Whether these modifications function synergistically or antagonistically to regulate RNA fate is largely unknown and requires further investigation. Additionally, with the discovery of an increasing number of modification sites, a critical question arises: how to distinguish functionally important modifications from background noise? From a biomedical perspective, it is essential to link RNA modifications with human disease. Identifying disease‐associated genetic mutations or SNPs that disturbs modifications will help elucidate their direct contributions to cancer pathology.

The progress of this field relies on high‐throughput sequencing technologies. While antibody‐based methods are widely used, they are often limited by cross‐reactivity and low resolution. Although single‐base resolution sequencing has been developed for major modifications, accurate detection of low‐abundance or rare modification types remains a great challenge. Future studies should focus on developing more sensitive technologies to capture these rare events without bias. Moreover, current bulk sequencing methods often obscure the heterogeneity and lose the spatial information of RNA modifications. Acquiring the spatial landscape at single‐cell resolution, namely, mapping exactly where modified RNAs are distributed within specific cell types and subcellular compartments, is crucial for understanding how these modifications function within the complex TME.

Finally, translating these biological findings into clinical treatments remains great challenges. It is difficult to develop potent and specific small‐molecule inhibitors targeting RNA‐modifying enzymes, especially in achieving precise modulation of the TME. Since RNA‐modifying enzymes are expressed in both tumor and normal cells, systemic inhibition may cause off‐target toxicity. Therefore, the challenge lies in how to effectively suppress tumor growth by remodeling the TME without compromising systemic immune function or normal tissue homeostasis. A promising strategy is to develop tissue‐specific delivery systems, such as nanoparticle‐encapsulated inhibitors or antibody‐drug conjugates, to precisely deliver the drug to the tumor site. Additionally, combinatorial approaches of RNA modification inhibitors with ICB or chemotherapy might enhance antitumor efficacy. By adopting these strategies, we can balance the therapeutic benefits against systemic risks.

## Author Contributions

**Qiwen Li**: conceptualization, original draft, writing – review and editing. **Yating Sun**: conceptualization, original draft, writing – review and editing. **Shuibin Lin**: supervision, funding acquisition, writing – review and editing. **Quan Yuan**: conceptualization, supervision, funding acquisition, writing – review and editing. All authors have read and approved the final manuscript.

## Ethics Statement

The authors have nothing to report.

## Conflicts of Interest

The authors declare no conflicts of interest.

## Data Availability

The data that support the findings of this study are available from the corresponding author upon reasonable request.
